# Multi-omics profiling reveals microbial regulation of a key aromatic ester phenethyl acetate formation in fermented alfalfa and its impact on sheep feed preference

**DOI:** 10.1016/j.fochx.2025.103249

**Published:** 2025-11-04

**Authors:** Zhihui Fu, Tianwei Wang, Jiaqi Zhang, Wenzhao Wang, Xiumin Zhang, Muhammad Tahir, Jin Zhong

**Affiliations:** a State Key Laboratory of Microbial Diversity and Innovative Utilization, Institute of Microbiology, Chinese Academy of Sciences, Beijing 100101, China; b School of Life Sciences, University of Chinese Academy of Sciences, Beijing 101408, China; c Key Laboratory for Agro-Ecological Processes in Subtropical Region, Institute of Subtropical Agriculture, Chinese Academy of Sciences, Changsha, Hunan 410125, China

**Keywords:** Fermented alfalfa, Flavor compounds, Metabolic pathway, Metagenomics, Feed intake

## Abstract

This study utilized headspace solid-phase microextraction coupled with gas chromatography–mass spectrometry (HS-SPME/GC–MS) to identify the key volatile flavor compounds in fermented alfalfa. The contribution of core microbiota to forming these key flavor compounds was investigated using a combination of absolute quantification of 16S rRNA gene copy number and metagenomic technology. Additionally, the critical roles of core fermentation microorganisms were quantitatively detected and validated through liquid chromatography mass spectrometry (LC-MS). Results revealed that *Lactiplantibacillus plantarum* B90 treated group achieved superior fermentation quality, with esters and aldehydes being the dominant volatile flavor compounds. Phenethyl acetate was the only aromatic ester that was significantly up-regulated after fermentation. The aryl alcohol dehydrogenase from *L. plantarum* facilitated the conversion of phenylacetaldehyde into phenylethyl alcohol, which serves as the precursor for phenethyl acetate. Furthermore, fermented alfalfa sprayed with phenethyl acetate was associated with increased feed intake in sheep. These findings propose new insights for microbial modulation of fermented flavor in fermented forage to enhance sheep feed intake.

## Highlights

• Phenethyl acetate was the only upregulated ester in LP-treated fermented alfalfa.
• Aryl alcohol dehydrogenase in L. *plantarum* is key for phenethyl acetate synthesis.
• Phenethyl acetate was associated with increased feed intake in sheep.

## Graphical abstract

**Figure:**
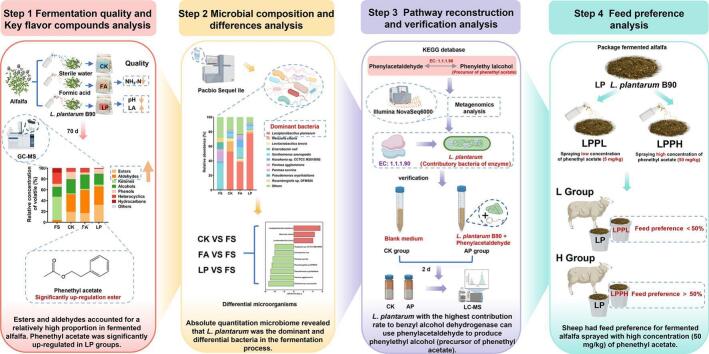

## 1 Introduction

Alfalfa (*Medicago sativa* L.) is known as the king of forages due to its high protein content and nutritional value, and it is the most widely cultivated legume forage crop worldwide. Fermentation improves its palatability and preservation, making fermented alfalfa a key component of ruminant diets (Tatli Seven et al., 2024). While most studies have focused on improving fermentation quality and microbial regulation (Liao et al., 2023; Wang, Chen, et al., 2019; Xia et al., 2024), limited attention has been given to the formation and regulation of flavor compounds, which are critical for feed intake and milk quality (Manzocchi et al., 2021; Zhang et al., 2021). Therefore, elucidating the role of core microorganisms of fermented alfalfa in the regulation of flavor compounds is crucial for maximizing its resource value and improving its utilization efficiency.

The flavor profiles of fermented products are mainly composed of volatile and non-volatile compounds, which are mainly produced by the metabolic activities of microorganisms, particularly lactic acid bacteria (LAB) (Wang et al., 2022). Additives can modify these microbial communities and thereby influence flavor development (Zhang et al., 2021). For instance, LAB inoculation can increase the aromatic compounds in fermented alfalfa (Liu, Du, Xie, et al., 2024), while chemical additives such as formic acid improve fermentation quality but their effects on flavor profiles remain unclear (Zhang et al., 2023). Although recent studies have examined flavor compounds and microbial associations in various fermented forages, such as stylos, oats and alfalfa (Liu, Du, Xie, et al., 2024; Zhang et al., 2021), the absolute quantification of microbial populations and their specific contributions to flavor formation are still poorly understood. Recent studies have demonstrated that microorganisms play key roles in regulating flavor formation in fermented foods (Xiong et al., 2024; Yang et al., 2021). However, despite increasing evidence from other fermented products, the mechanisms by which microorganisms influence flavor compound profiles in fermented alfalfa remain largely unexplored. Metabolomic approaches have begun to reveal links between fermentation metabolites and feed preference (Scherer et al., 2021), yet the roles of key flavor compounds in regulating feed intake remain to be clarified.

Fermentation is primarily driven by microorganisms, whose community structure and function critically determine flavor formation (Xiong et al., 2024). Variations in microbial composition caused by different additives markedly influence the sensory characteristics of fermented products (Nguyen et al., 2022). However, most studies (Liu, Du, Xie, et al., 2024; Xiong et al., 2024) have relied on relative abundance data, which may not accurately reflect true microbial dynamics (Rao et al., 2021). Absolute quantification of 16S rRNA sequencing provides a more precise understanding of microbial population changes during fermentation. In addition, headspace solid-phase microextraction coupled with gas chromatography–mass spectrometry (SPME/GC–MS) and Ultra-high-performance liquid chromatography-mass spectrometry (UHPLC-MS/MS) has been widely used in the detection of volatile and non-volatile flavor compounds such as cheese (Ricci et al., 2023), and meat (Man et al., 2023). Metagenomic sequencing provides extensive microbial genomic information, which helps to reveal the mechanisms of microorganisms contributing to the formation of flavor compounds (Liu et al., 2019). This technology has been widely used in the research of fermented products such as *daqu* (Yang et al., 2021), *Huangjiu* (Yu et al., 2024), and fermented vegetables (Xiong et al., 2024).

Therefore, this study aimed to investigate the changes in the microbial community of fermented alfalfa treated with different additives and to further clarify how microorganisms regulate the formation of characteristic flavor compounds. In particular, we verified the important role of characteristic flavor substances in combination with the feeding behavior of sheep. Our results open a new way and provide a theoretical basis for developing high-quality flavor fermented forage additives and ruminant attractants.

## 2 Materials and methods

### 2.1 Raw materials and fermented alfalfa preparation

Alfalfa third crop (at the booting stage) was harvested on July 26th, 2023, at Lingshou County, Shijiazhuang Province, China (38°30′ N; 114°37′ E). The harvested materials were wilted to approximately 35 % dry matter (DM) and then cut into 2 cm lengths. The chopped forage was then inoculated with *Lactiplantibacillus plantarum* B90 (10^6^ CFU/g fresh matter, FM) as the LP group, treated with formic acid (5 mL/kg FM) as the FA group, and left untreated as the natural fermentation (CK) group. *L. plantarum* B90 (CGMCC No. 13318) was isolated from fermented sweet sorghum. A total of 18 bags (3 treatments × 6 replicates) were prepared and stored at room temperature (28 ± 2 °C). After 70 days of fermentation, samples were collected and their nutritional quality, fermentation quality, volatile flavor compounds, non-volatile flavor compounds, and microbial communities were analyzed.

The baled fermented alfalfa used in the feeding experiment was sourced from the first cutting of alfalfa (at the booting stage), harvested on June 30, 2024 at the Hadatu Ranch in Hulunbeier, Inner Mongolia Autonomous Region, China (49°27′26″ N; 120°17′49″ E). The harvested material was wilted to approximately 35 % DM and chopped into 2 cm lengths. The chopped grass was inoculated with L. *plantarum* B90 (10^6^ CFU/g FM) and fermented for 352 days to produce the control group (LP) for the feeding experiment. A low concentration (5 mg/kg) of phenethyl acetate was uniformly sprayed onto the LP group to form the test group LPPL, while a high concentration (50 mg/kg) was applied to establish the test group LPPH.

### 2.2 Analysis of fermentation and nutrition quality

Fermentation quality, including pH value, lactic acid (LA) content, acetic acid (AA) content, and ammonia nitrogen (NH_3_−N) content, was determined according to the methods of Zhang et al. (2021). Nutrition quality, including the DM content, crude protein (CP) content, neutral detergent fiber (NDF) content, acid detergent fiber (ADF) content, and water-soluble carbohydrate (WSC) content. The WSC content was determined according to the methods of Wang, Chen, et al. (2019). The DM content was determined by oven drying at 65 °C for 48 h. The CP content was measured using a Kjeldahl apparatus (Gerhart Vapodest 50 s, Germany) following the method described by Patrica (1997). The NDF and ADF were quantified using the procedures outlined by Van et al. (1991), with an Ankom A2000i fiber analyzer (A2000i, Ankom Technology, Macedon, NY, United States).

### 2.3 Analysis of flavor compounds

After fully mixed, 10 g of uniform and representative fermented alfalfa samples were collected, immediately frozen in liquid nitrogen, and stored at −80 °C until analysis. The volatile flavor compounds were detected by gas chromatography and high-resolution orbital spectroscopy combined with headspace solid-phase microextraction (HS-SPME), which was slightly modified according to the methods of Yang, Li, et al. (2022). GC–MS data were processed via Xcalibur 4.1 and TraceFinder 4.0 software (Thermo Scientific). Aroma compounds were identified by mass spectrometry and linear retention indices, referencing NIST17 (v2.3) and domestic libraries (Yang, Li, et al., 2022). Additionally, volatile flavor compounds were semi-quantitatively analyzed according to peak area relative to the internal standard, 2-nonanol. Characteristic volatile flavor compounds were calculated via the method of Xiong et al. (2024).

The non-volatile flavor compounds were analyzed by LC-MS/MS on a Thermo UHPLC-Q Exactive HF-X system equipped by Majorbio Bio-Pharm Technology Co., Ltd. (Shanghai, China), following the procedure described by Fu et al. (2022). UHPLC–MS/MS analysis was conducted based on the method established by Yang, Wang, et al. (2022). Based on the variable weight value (VIP) obtained by the orthogonal partial least squares discriminant analysis (OPLS-DA) model and the *P* value of the student's *t*-test, VIP > 1 and *P* < 0.05 were used as screening conditions to analyze the differential compounds between fermented alfalfa treated with different additives and fresh alfalfa samples.

### 2.4 Absolute quantification of 16S rRNA gene sequencing data

DNA was extracted from the sample following the method described by Huang et al. (2023). Briefly, 10 g of the sample was shaken with sterile PBS for 10 min. The resulting mixture was then filtered through four layers of coarse cotton cloth. The filtrate was subsequently centrifuged at 10,000 rpm and 4 °C for 10 min (min) to collect the sediment for DNA extraction. Microbial DNA was extracted from samples via the FastDNA™ Spin Kit for Soil (MP Biomedicals, Solon, OH, US) according to the manufacturer's instructions. 16S sequencing was conducted by Majorbio Bio-Pharm Technology Co., Ltd. (Shanghai, China) to determine absolute quantitative microbial abundance. PCR amplification, Illumina data processing, and functional analysis followed the methods of Tan et al. (2021).

### 2.5 Metagenomic sequencing analysis

DNA purity was assessed, and paired-end (PE) libraries were constructed following established protocols or instructions (Yang et al., 2021). Metagenomic sequencing was performed on an Illumina NovaSeq (Illumina, US) platform, with analysis completed by Majorbio Bio-Pharm Technology Co., Ltd. (Shanghai, China). Data quality control, sequence optimization, gene prediction, and nonredundant gene set construction were performed as described by Yang et al. (2021). High-quality reads were aligned to nonredundant gene sets (with 95 % identity) via SOAPaligner (version 2.21) to quantify gene abundance. The RPKM method is used to normalize the single gene abundance by calculating the number of reads per thousand bases per million reads sequenced (Lawson et al., 2017). Each functional gene was taxonomically assigned to its most likely host species (e.g., *L. plantarum*) based on BLAST alignment results. The relative contribution of each species to enzymatic function was then calculated by weighting the normalized gene-level RPKM values.

### 2.6 Metabolic pathway validation

The blank liquid MRS medium was used as the control group (CK), and the liquid MRS medium with phenylacetaldehyde was used as the experimental group (AP). The AP group was inoculated with L. *plantarum* B90 and incubated at 37 °C for 48 h. Concentrations of phenylacetaldehyde and phenylethyl alcohol were then quantified via LC-MS, using the protocols established by Liu, Du, Sun, et al. (2024).

### 2.7 Animals, feeding management and experimental design

All animal procedures used in this study were reviewed and approved by the Animal Care Committee (approval number: ISA-2025-0041), Institute of Subtropical Agriculture, Chinese Academy of Sciences. The experiment was carried out in the Yellow River Estuary Tan Sheep Institute of Industrial Technology (Lijin County, Dongying City, Shandong Province, China). Twelve healthy 7-month-old sheep with no significant difference in body weight were selected. The sheep were randomly assigned to two experimental groups (*n* = 6 per group): one group received LPPL-treated fermented alfalfa as the test diet, and the other received LPPH-treated fermented alfalfa. All animals were housed individually in pens equipped with a double-bin feeding system for assessing feed preference, a method adapted from Scherer et al. (2019). In this design, two separate feed bins were placed at the feeding inlet of each pen. For each sheep, one bin contained the control diet (LP-fermented alfalfa), while the other bin contained the respective test diet (either LPPL-fermented alfalfa or LPPH-fermented alfalfa, depending on the group assignment). Feed intake from each bin was recorded to evaluate palatability and preference. Each barrel was loaded with 1000 g samples, and feed was offered twice daily at 06:00 and 16:00. During each feeding session, both feed bins were made available simultaneously for a 30-min access period, during which the animal could freely choose between the two fermented alfalfa types. This short-term, two-choice paradigm was used to evaluate initial intake preference (Scherer et al., 2021). After 30 min of feeding, residual feed in each barrel was collected and weighed to calculate intake. The total intake for each fermented alfalfa type per day was calculated as the sum of the amounts consumed during the morning and evening 30-min sessions. The feed intake over the two sessions was used as the basis for calculating feeding preference, defined as the proportion of intake from the experimental group relative to total intake (i.e., control group feed intake + test group feed intake) (Guo et al., 2022). The feed preference refers to the following formula:

$$\text{Feed preference}=\frac{\text{Test group feed intake}}{\left(\begin{array}{c} \text{Control group feed intake}\\ +\text{Test group feed intake} \end{array}\right)}\times 100\%$$

here: feed preference ranges from 0 to 100%; when feed preference is less than 50 %, sheep tend to feed on the control group fermented alfalfa; when feed preference exceeds 50 %, sheep are more inclined to feed on the test group fermented alfalfa; when feed preference equals 50 %, sheep show no preference between the control group and test group fermented alfalfa.

After feeding fermented alfalfa, the animals were then fed a total mixed ration composed of fermented corn and grain mixture to meet the nutritional needs of the animals. The pre-trial period was 7 days, and the formal feeding period was 8 days. During the experiment, all animals were given equal access to feed and free water to ensure that all animals received the same nutritional level and management conditions.

### 2.8 Statistical analysis and visualization

Statistical analysis was performed using IBM SPSS Statistics 27 (R27.0.1.0 64). One-way ANOVA was employed to evaluate nutritional and fermentation quality. The *t*-test was used to compare the contents of phenylacetaldehyde and phenylethyl alcohol among the different treatments, and data visualization was performed via GraphPad Prism 10 (v10.0.2). A linear mixed-effects model was employed to analyze feed intake, average DM intake, feed preference, feeding rate, and intake rate in sheep, with results visualized using R (v4.3.0) (Guo et al., 2022). Linear mixed-effects models were fitted using restricted maximum likelihood (REML) in R (v4.3.0). Multiple comparisons were adjusted using the false discovery rate (FDR) method (*P* < 0.05). Effect sizes (Cohen's d) and 95 % confidence intervals are shown in the figures. Bioinformatics analyses, including Veen analysis, Partial Least Squares Discriminant Analysis (PLS-DA), Orthogonal Partial Least Squares Discriminant Analysis (OPLS-DA), alpha diversity, Principal Coordinate Analysis (PCoA), and Linear Discriminant Analysis (LDA), were performed via OmicStudio (https://www.omicstudio.cn/tool). Metabolic pathway analysis involved comparing the amino acid sequence of the nonredundant gene set with that of the KEGG database (https://www.genome.jp/kegg/) to determine gene functions and produce bacteria, which were visualized via GraphPad Prism 10 (v10.0.2) (Yang et al., 2021).

## 3 Results and discussion

### 3.1 Effects of different additives on the quality and key flavor compounds of fermented alfalfa

**Fig. 1:**
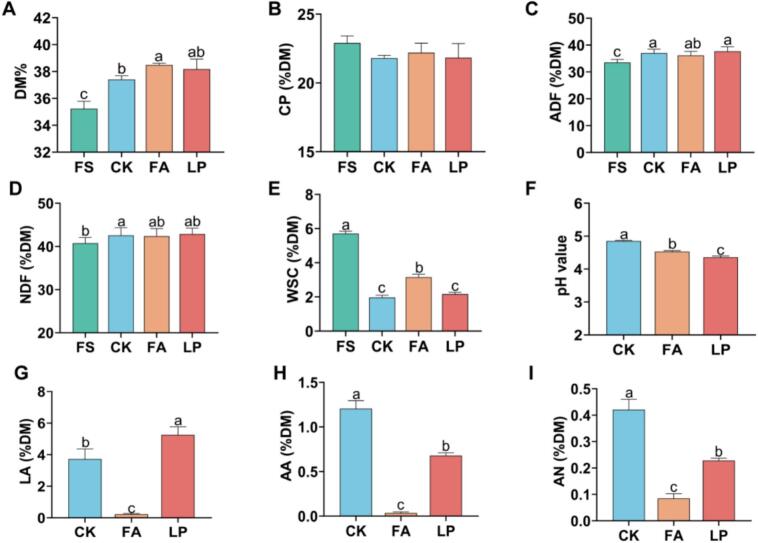
**Chemical composition and fermentation characteristics of fermented alfalfa with different additives.** (A) the dry matter (DM) content of fermented alfalfa. (B) the crude protein (CP) content of fermented alfalfa. (C) the acid detergent fiber (ADF) content of fermented alfalfa. (D) the neutral detergent fiber (NDF) content of fermented alfalfa. (E) the water-soluble carbohydrate (WSC) content of fermented alfalfa. (F) the pH values of fermented alfalfa. (G) the lactic acid (LA) content of fermented alfalfa. (H) the acetic acid (AA) content of fermented alfalfa. (I) the ammonia nitrogen (NH_3_-N) content of fermented alfalfa. Different lowercase letters indicate significant differences (*P* < 0.05). FS: fresh samples of alfalfa; CK: sterilized water; FA: formic acid; LP: *Lactiplantibacillus plantarum* B90.

Fermented alfalfa is a key feed resource for ruminants; its quality and flavor significantly affect palatability and feed intake. Understanding changes in quality and flavor compounds before and after fermentation is crucial for optimal resource utilization. In this study, nutritional quality, including the contents of CP, ADF, and NDF, did not significantly differ among the treatment groups, but the additives reduced the consumption of WSC. This could be attributed to the fact that LAB mainly consumes soluble carbohydrates as their main fermentation substrates during growth and reproduction (Huang et al., 2023). However, in terms of fermentation quality, the LP group exhibited significantly (*P* < 0.05) lower pH value (4.36) and higher LA content (5.26 % DM) than that of the CK and FA groups. Additionally, the FA group exhibited significantly lower AN and AA contents (*P* < 0.05) compared to the CK and LP groups (Fig. 1). These results indicate that both FA and LP have minimal effects on nutritional quality, but they can improve fermentation quality. Specifically, formic acid may reduce protein degradation, whereas L. *plantarum* may increase LA fermentation. Notably, the LP group exhibited the lowest pH value and the highest LA content, suggesting that its fermentation quality was superior compared to the other groups. According to Scherer et al. (2021), the feed intake of alfalfa and red clover mixed fermented products inoculated with LAB was significantly higher than that of the control group. These findings suggest that the addition of LAB during fermentation may enhance feed intake in ruminants.

**Fig. 2:**
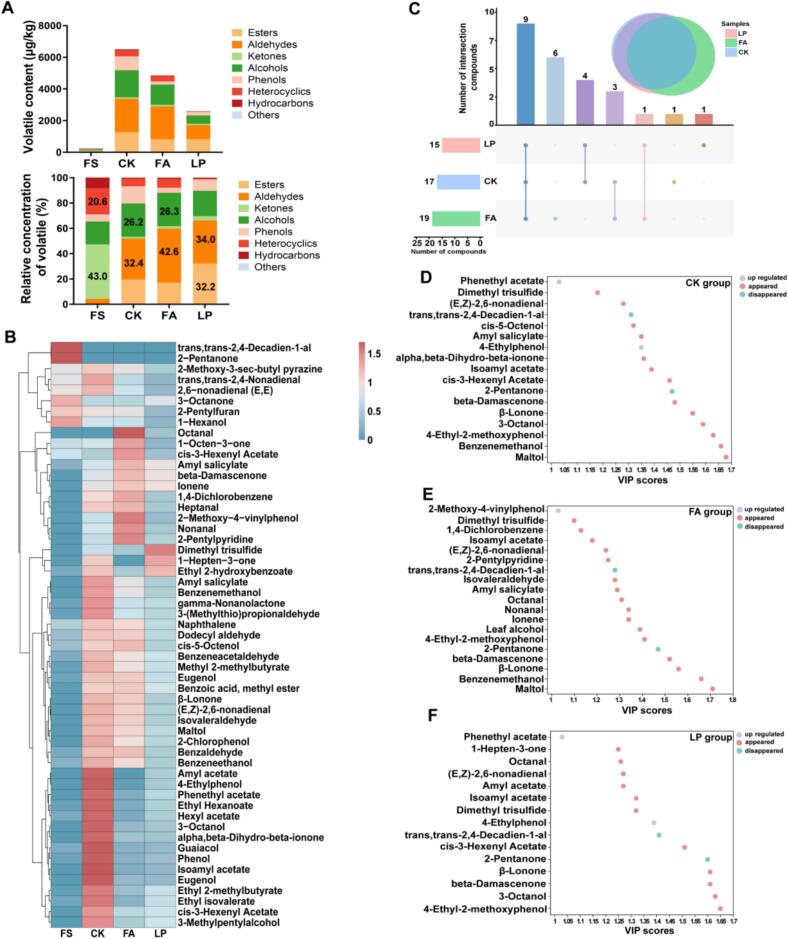
**Characteristics of key volatile flavor compounds in fermented alfalfa with different additives.** (A) Contents of different types of key flavor compounds; relative concentrations of different types of key volatile compounds. (B) Clustering heatmap analysis based on data standardization. (C) Venn analysis of key flavor compounds. The key flavor compounds ranked by variable importance in projection (VIP) of CK group (D), FA group (E), and LP group (F) based on the partial least squares discriminant analysis (PLS-DA) model. FS: fresh samples of alfalfa; CK: sterilized water; FA: formic acid; LP: *Lactiplantibacillus plantarum* B90.

Volatile flavor compounds play a vital role in determining the quality attributes of fermented products, as they significantly influence the overall sensory quality. Additionally, the fermentation process also altered the composition of flavor compounds in fermented alfalfa. Compared with fresh samples (FS), the total amount of characteristic volatile flavor compounds (OAV ≥ 1) increased after fermentation, significantly enhancing the overall flavor (Xiong et al., 2024). Different additive treatments resulted in distinct aroma profiles. In the FS group, ketones (102.39 μg/kg) and heterocyclic compounds (49.11 μg/kg) were the most abundant, accounting for 63.6 % of the total compounds. In the LP group, the relative contents of esters and aldehydes were the highest, accounting for 66.2 % of the total compounds. In the FA and CK groups, the relative contents of aldehydes and alcohols were the highest, accounting for 68.9 % and 58.6 %, respectively (Fig. 2A). The FA group comprises primarily aldehydes and alcohols, and the LP group is dominated by esters and aldehydes. Volatile esters and aldehydes are major contributors to pleasant aromas such as fruity, sweet, and floral notes in fermented products (Yao et al., 2023; Zhao et al., 2021).Owing to their higher volatility compared with acids and alcohols, these compounds can rapidly diffuse and dominate the overall aroma profile during short exposure periods (Hafner et al., 2013). This suggests that the LP group may volatilize more esters and aldehydes with aromatic or fruity flavors in a short time, potentially increasing the palatability and feed intake of animals. Similarly, Liu, Du, Xie, et al. (2024). reported that terpenoids, esters, and heterocyclic compounds were the main volatile components in fermented alfalfa, likely due to differences in additive types. After fermentation, two flavor compounds disappeared and 53 characteristic flavor compounds appeared (Fig. 2B). This highlights that fermentation altered the composition of flavor compounds, leading to increased aroma production in fermented alfalfa. PLS-DA analysis was performed to identify key flavor compounds that significantly changed across the different additive groups. The various treatment groups exhibited a differing number of key flavor compounds, with a total of 9 distinct aroma substances identified (Fig. 2C). Among the different groups, phenethyl acetate (FC = 204.55) was notably up-regulated in the CK group (Fig. 2D), and 2-methoxy-4-vinylphenol (FC = 80.72) was notably up-regulated in the FA group (Fig. 2E), whereas phenethyl acetate (FC = 75.97) and 4-Ethylphenol (FC = 2540.8) were significantly up-regulated in the LP group (Fig. 2F). Of the two compounds significantly up-regulated in the LP group, phenethyl acetate is the only aromatic ester, whereas 4-Ethylphenol is a phenolic compound. These differences in chemical class may contribute to distinct olfactory properties and potential effects on feed preference.

**Fig. 3:**
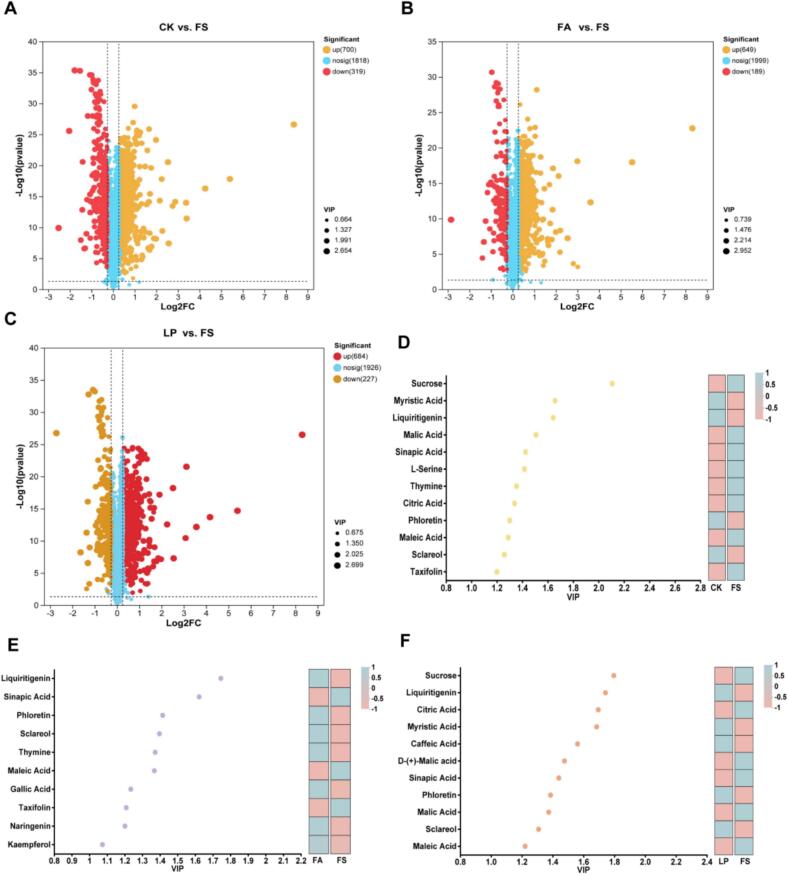
**Characteristics of non-volatile compounds in fermented alfalfa with different additives.** (A) Volcano plot showing the numbers of differentially expressed non-volatile compounds between the CK and FS groups. (B) Volcano plot showing the numbers of differentially expressed non-volatile compounds between the FA and FS groups. (C) Volcano plot showing the numbers of differentially expressed non-volatile compounds between the LP and FS groups. The key taste compounds ranked by variable importance in projection (VIP) of CK group (D), FA group (E), and LP group (F) based on PLS-DA model. The color box on the right represents the relative concentration of metabolites in different groups. FS: fresh samples of alfalfa; CK: sterilized water; FA: formic acid; LP: *Lactiplantibacillus plantarum* B90.

Apart from volatile flavor compounds, non-volatile compounds play a key role in fermented forage palatability, particularly those that are associated with the taste. After fermentation, the levels of differential non-volatile compounds between different treatment groups and FS changed greatly (Fig. 3A-C). In the CK group, we identified 700 non-volatile compounds that were significantly up-regulated and 319 non-volatile compounds that were significantly down-regulated, including 12 compounds related to taste (Fig. 3D). In the FA group, we identified 649 non-volatile compounds that were significantly up-regulated and 189 non-volatile compounds that were significantly down-regulated, including 10 compounds related to taste (Fig. 3E). In the LP group, we identified 684 non-volatile compounds that were significantly up-regulated and 227 non-volatile compounds that were significantly down-regulated, including 11compounds related to taste (Fig. 3F). Malic acid (FC = 0.71) and l-serine (FC = 0.71) were significantly down-regulated in the CK group. Thymine (FC = 1.26) and gallic acid (FC = 1.27) were significantly up-regulated in the FA group and caffeic acid (FC = 1.43) was significantly up-regulated in the LP group. The VIP value of sucrose in the CK and LP groups was higher compared to the FA group, but it was significantly lower than that of FS. This may be due to the use of LAB as a necessary carbon source for growth during fermentation. Sucrose is used as an additive and significantly improved the quality of fermented mulberry leaf (Wang, He, et al., 2019). Liquiritigenin (FC = 1.43) had the highest VIP value in the FA group, which was significantly up-regulated in the FA group. It is a non-sugar natural sweetener, which is one of the natural sweeteners currently used to adjust the taste of food (Keranmu et al., 2022). These characteristics may have an impact on the palatability of fermented forage.

### 3.2 Effects of different additives on microbial community in fermented alfalfa

**Fig. 4:**
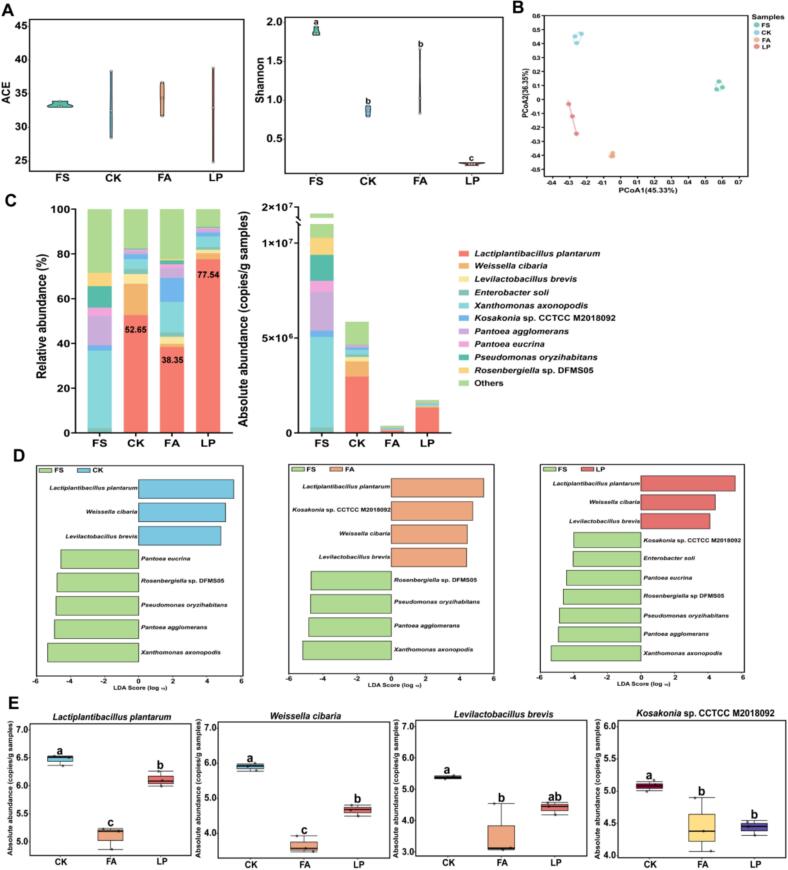
**Microbial composition and variations in fermented alfalfa under different additive treatments.** (A) Alpha diversity indices, including the ACE index and Shannon index, in different groups. (B) Principal coordinate analysis (PCoA) of different additive groups. (C) The relative abundance and absolute abundance of microbiota in different additive groups at the species level. (D) Linear discriminant analysis (LDA) of different additive groups with fresh materials. (E) Analysis of the differences in core microorganisms in different groups. Different lowercase letters indicate significant differences (*P* < 0.05). FS: fresh samples of alfalfa; CK: sterilized water; FA: formic acid; LP: *Lactiplantibacillus plantarum* B90.

Forage fermentation is a dynamic process driven by microbial succession and metabolite changes. The microbial community structure influences metabolic pathways, whereas alterations in metabolic compounds may impact microbial activity. In this study, there were no significant differences in the ACE index between treatments, whereas the LP group had a lower Shannon index compared to the other groups (Fig. 4A). Significant variations were observed in the microbial community structure (Fig. 4B) and composition (Fig. 4C) among the treatment groups. The L. *plantarum* was the dominant species in the LP and CK groups compared to the FA group. The LP group had a lower proportion of L. *plantarum* with 1.33 × 10^6^ copies/g of samples compared to the CK group (2.97 × 10^6^ copies/g samples), but it was higher than the FA group (1.32 × 10^5^ copies/g samples). Additionally, the total absolute abundance of microbial community was higher in the CK group compared to the FA and LP groups, highlighting that additive reduced the total absolute abundance of microbial community by simplifying it. The application of additive promotes LA accumulation, which rapidly lowers pH and suppresses the growth of spoilage organisms. This results in accelerated acidification, improved fermentation efficiency and stability, and higher overall silage quality. This finding was consistent with the results of *Sesbania cannabina* and sweet sorghum mixed fermented products (Xia et al., 2024). The reduction in metabolic activity of LAB could be attributed to two primary factors: the altered chemical composition of the fermented forage and competition among different LAB strains for substrates. The addition of L. *plantarum* in the LP group might have resulted in the rapid proliferation of L. *plantarum*, which significantly enhanced the LA production. This increase in LA content led to a decrease in pH value, effectively inhibiting the growth of undesirable microorganisms, as well as impacting the L. *plantarum* abundance at the end of ensiling. Consequently, this inhibition may explain why the absolute abundance of L. *plantarum* in the LP group was lower at the end of fermentation compared to the CK group. Although each group was dominated by L. *plantarum* after fermentation, the relative abundance of L. *plantarum* in the LP group (77.54 %) was the highest compared to the others.

We conducted a multilevel species difference analysis using LEfSe to compare species differences among treatment groups and FS (Fig. 4D). The biomarkers of the CK and LP groups were L. *plantarum*, *W. cibaria*, and *Levilactobacillus brevis.* The biomarkers of the FA group were L. *plantarum*, *W. cibaria*, *Levilactobacillus brevis,* and *Kosakonia* sp. CCTCC M2018092. *L. plantarum* and *W. cibaria* were more abundant in the LP group than in the FA group (Fig. 4E). These biomarkers may play an important role in forming metabolites in fermented alfalfa and represent potential core functional microbiota taxa. Liu, Du, Xie, et al. (2024). reported that the microbial functions in fermented alfalfa were significantly enriched in carbohydrate and amino acid metabolism, biosynthesis of secondary metabolites, and degradation of aromatic compounds. In this study, the core microorganism L. *plantarum* plays a key role in the metabolism of organic acids and esters by participating in the biosynthesis of LA and AA, and in the production of phenethyl acetate, a key volatile ester compound derived from phenylalanine metabolism. The previous study (Liao et al., 2023) highlighted that the antibacterial and acid production capabilities of L. *plantarum* substantially enhance microbial regulation, optimize the fermentation environment, and improve product quality. In addition, *L. plantarum* converts carbohydrates into LA via the Embden-Meyerhof pathway, which in turn contributes to the formation of flavor compounds. This microorganism is rich in flavor-promoting enzymes and serves as an effective starter for fermented dairy and vegetable products (Liang et al., 2023). *W. cibaria* exhibits notable probiotic properties and strong proteolytic activity which are crucial for the fermentation process. The proteolytic enzymes released by such bacteria play crucial roles by releasing peptides and amino acids from casein, which substantially influence the aroma, texture, and taste of the final product. According to Fan et al. (2022), the fermentation of binary probiotics can enhance the production of volatile flavor compounds, such as 2-heptanone, naphthalene, hexanal, and 1-pentanol, by *L. brevis*. Additionally, the synergistic relationships between microorganisms can further impact flavor formation (Liang et al., 2023). Meanwhile, specific effects of *Kosakonia* sp. CCTCC M2018092, known for producing fucose-rich exopolysaccharide, on flavor substances remains to be explored.

**Fig. 5:**
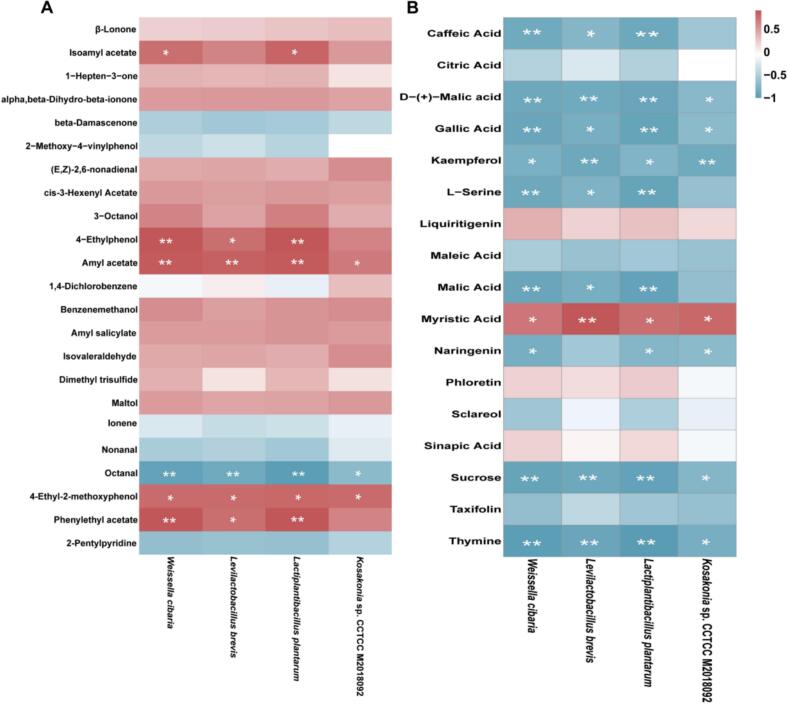
**Correlation analysis between important flavor compounds and differential microorganisms based on Spearman correlation coefficient.** (A) Correlation analysis between differential microorganisms and characteristic volatile flavor compounds. (B) Correlation analysis between differential microorganisms and significant changes in important non-volatile flavor compounds. The color type identifies positive (red) or negative (blue) correlations. * *p* < 0.05, ** *p* < 0.01. (For interpretation of the references to color in this figure legend, the reader is referred to the web version of this article.)

To further explore the regulation of microorganisms on flavor compounds, we conducted a Spearman correlation analysis to examine the relationships between characteristic volatile and non-volatile flavor substances, and biomarkers (Fig. 5A and B). The biomarkers L. *plantarum* and *W. cibaria* were significantly (*P* < 0.01) positively correlated with 4-Ethylphenol, Amyl acetate, and 4-Ethyl-2-methoxyphenol, while significantly (*P* < 0.01) negatively correlated with octanal (Fig. 5A). It is worth noting that L. *plantarum*, *L. brevis*, and *W. cibaria* were significantly (*P* < 0.01) positively correlated with phenethyl acetate, indicating that these three strains may synergistically enhance its production. In addition, the core microorganisms L. *plantarum* and *W. cibaria* were significantly (*P* < 0.05) positively correlated with myristic acid, whereas showed substantial negative association (*P* < 0.01) with caffeic acid, gallic acid, l-serine, malic acid, sucrose, and thymine. Although the correlation between key flavor compounds and differential microorganisms has been explored, the detailed mechanism through which microorganisms contribute to the flavor compound formation, as well as their dependence on metabolic pathways and related enzymes, remain to be further elucidated.

### 3.3 Metagenomic analysis of metabolic pathways for key flavor compounds in fermented alfalfa

**Fig. 6:**
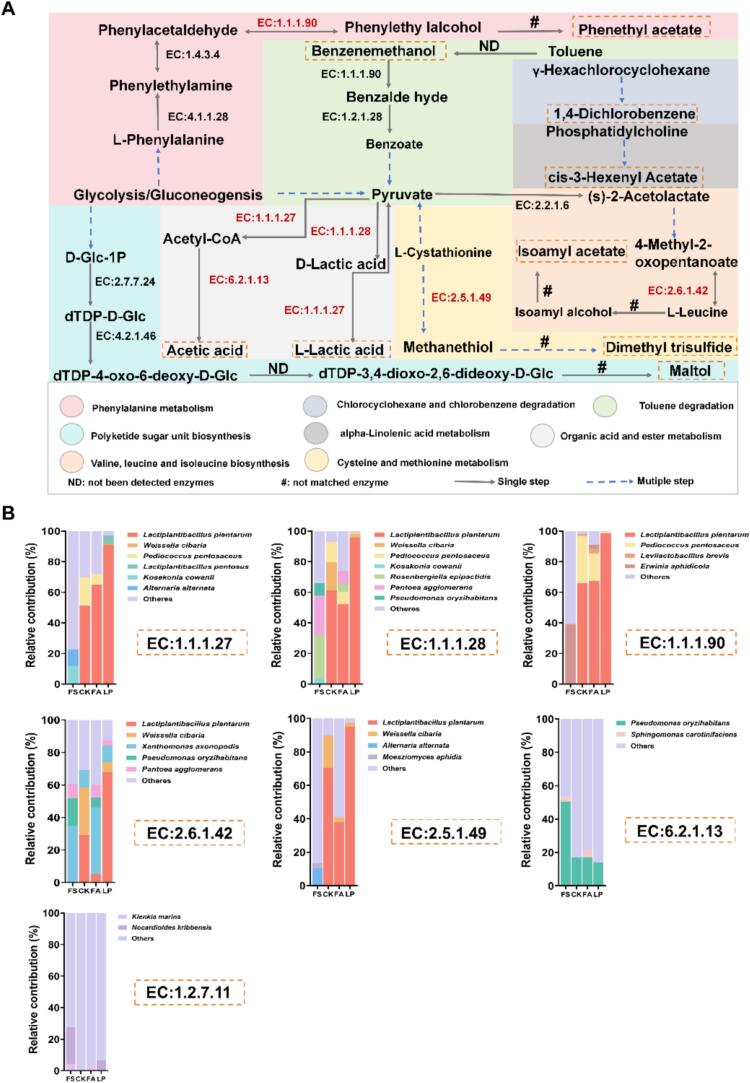
**The metabolic network of characteristic flavor compounds and the contribution of core microorganisms to the formation of characteristic flavor compounds.** (A) Formation pathway of characteristic flavor compounds. The color of the region represents different flavor compound formation or substrate utilization pathways. The characteristic flavor compounds are expressed in yellow boxes and key enzymes are expressed in red. EC:1.1.1.27, L-lactate dehydrogenase; EC:1.1.1.28, D-lactate dehydrogenase; EC:1.1.1.90, aryl-alcohol dehydrogenase; EC:2.6.1.42, branched-chain amino acid aminotransferase; EC:2.5.1.49, *O*-acetyl-L-homoserine sulfhydrolase; EC:6.2.1.13, acetyl-CoA synthetase; EC:1.2.7.11, 2-oxoacid oxidoreductase (ferredoxin); (B) Analysis of the contribution of microorganisms to key enzymes in different groups. FS: fresh samples of alfalfa; CK: sterilized water; FA: formic acid; LP: *Lactiplantibacillus plantarum* B90. (For interpretation of the references to color in this figure legend, the reader is referred to the web version of this article.)

To further analyze the formation mechanism of flavor compounds in fermented alfalfa, we reconstructed the metabolic pathways of LA, AA, and key flavor compounds using the KEGG database, which identified a total of eight distinct pathways (Fig. 6A). We integrated key enzyme data with metagenomic information to predict the contributions of key microorganisms and analyzed the formation mechanisms of these flavor compounds (Fig. 6B). We found that primary substrates (mainly WSC) are metabolized through glycolysis to produce key precursors for flavor compounds. Notably, LA and AA are the crucial important taste-active compounds of fermented vegetables and their formation mainly predominantly occurs through the glycolysis pathway, with pyruvate serving as the core metabolite (Xiong et al., 2024). Pyruvic acid is converted into LA by D-lactate dehydrogenase (EC1.1.1.28) and L-lactate dehydrogenase (EC1.1.1.27) enzymes, which are produced primarily by L. *plantarum*. Pyruvate is also converted to acetyl-CoA by 2-oxoacid oxidoreductase (EC1.2.7.11) enzyme, subsequently leading to the formation of AA by acetyl-CoA synthetase (EC6.2.1.13) enzyme. The organic acids produced by LAB are crucial for imparting the characteristics of sourness and odor of fermented products. These compounds interact with esters, aldehydes, and alcohols formed during fermentation to create the distinctive sour aroma of fermented alfalfa, which is similar to fermented vegetables (Liang et al., 2023). LA and AA are primary sources of sourness in fermented alfalfa, and the sour aroma produced by these compounds can stimulate livestock appetite, thereby encouraging increased consumption (Liu, Du, Xie, et al., 2024).

**Fig. 7:**
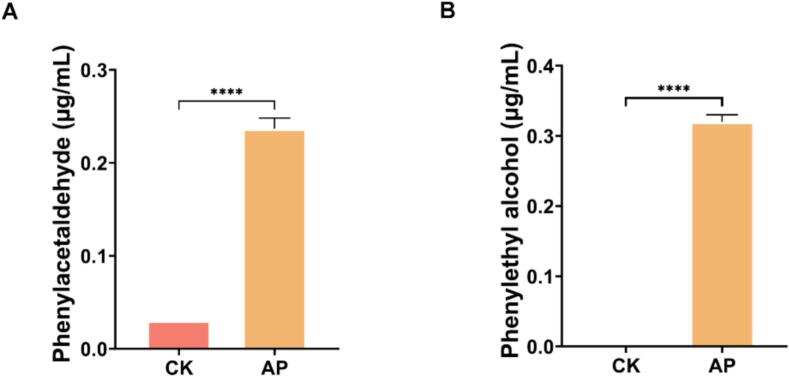
**Changes in key flavor compounds after in vitro fermentation.** (A) Content of phenylacetaldehyde in the fermentation broth of *Lactiplantibacillus plantarum* B90; (B) Content of phenylethyl alcohol in the fermentation broth of *Lactiplantibacillus plantarum* B90. CK: MRS liquid media; AP: MRS liquid media added phenylacetaldehyde; **** represent the significance between groups at probability levels of 0.0001.

It is well known that esters significantly influence the flavor of fermented forage (Scherer et al., 2021). Among the esters significantly up-regulated after fermentation in the LP group, only phenethyl acetate was identified. This indicated that phenethyl acetate was the key flavor ester. It is formed through the phenylalanine metabolic pathway where the precursor phenylethyl alcohol is produced by the action of aryl alcohol dehydrogenase (EC1.1.1.90) enzyme, which is produced by L. *plantarum* (with a strain contribution rate of 98.60 %). Furthermore, the formation of key volatile esters in fermented alfalfa involves both biochemical and abiotic processes, highlighting the complexity of flavor development during fermentation. Liu and Siezen (2006) demonstrated that LAB produces esters via acyltransferase and esterase activities, whereas nonbiological and nonenzymatic esterification occurs between carboxylic acids and alcohols at room temperature. The acidic fermentation conditions contribute to the esterification reaction (Hafner et al., 2013). Phenylethyl alcohol, characterized by a rose aroma, serves as the precursor of phenethyl acetate. During fermentation, it is esterified with AA in the fermentation environment to form phenethyl acetate, which imparts delicate flavors of ‘flower’, ‘fruit’, and ‘honey’ (Zhang et al., 2020). The results of in vitro experiments of L. *plantarum* B90 showed that the phenylacetaldehyde and phenylethyl alcohol contents in the AP group were significantly (*P* < 0.05) higher than those in the CK group (Fig. 7A-B). This highlights that L. *plantarum* B90 can use phenylacetaldehyde to synthesize phenylethyl alcohol. Phenethyl acetate is a high-value chemical utilized as both a food and flavoring ingredient (Sekar et al., 2022), which imparts fruity and floral odors for pickled chayote (Xiao et al., 2020). Based on this, we speculate that phenethyl acetate contributes to the flavor profile of fermented alfalfa, which may potentially increase the animal feed intake. However, this speculation requires further validation through animal feeding experiments.

### 3.4 Effects of phenethyl acetate sprayed fermented alfalfa on the feed behavior of sheep

Phenethyl acetate has sensory characteristics such as ‘flower’, ‘fruity’ and ‘honey’. In view of the fact that the initial feeding preference of ruminants is greatly affected by the volatile odor of feed (Scherer et al., 2019), we speculate that it may regulate feeding behavior, but there is no direct evidence. To this end, this study evaluated the short-term feeding response of sheep to high-quality alfalfa silage after exogenous spraying of different concentrations of phenethyl acetate.

**Fig. 8:**
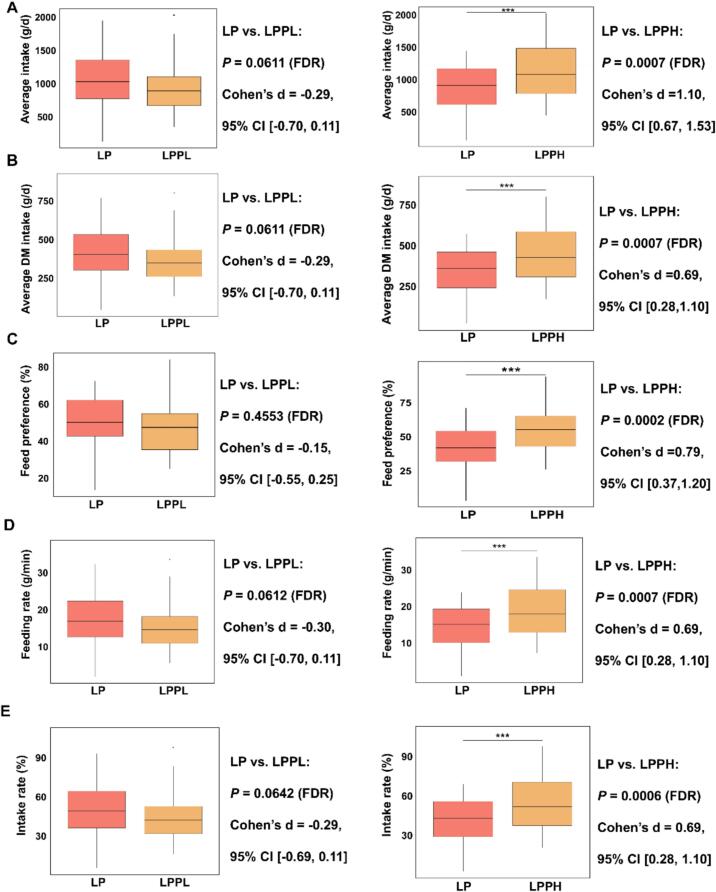
**Sheep feeding behavior on fermented alfalfa treated with varying phenethyl acetate levels.** (A) Average intake of sheep across experimental group; (B) Average DM intake of sheep across experimental group; (C) Feed preference of sheep across experimental group; (D) Feeding rate of sheep across experimental group; (E) Intake rate of sheep across experimental group. LP: *Lactiplantibacillus plantarum* B90; LPPL: low concentration of phenethyl acetate was sprayed on the fermented alfalfa in the LP group; LPPH: high concentration of phenethyl acetate was sprayed on the fermented alfalfa in the LP group; **** represents the significance between groups at probability levels of 0.0001. Statistical significance was assessed using linear mixed-effects models with restricted maximum likelihood (REML) estimation and post-hoc comparisons adjusted by false discovery rate (FDR) correction. Effect sizes are expressed as Cohen's d, where negative values indicate reduced preference.

The fermented alfalfa used in the feeding experiment was also fermented by adding L. *plantarum* B90. The 352-day fermented alfalfa (Supplemental Table 1) exhibited comparable nutritional and fermentation profiles to the 70-day fermented alfalfa, and both were classified as high-quality fermented feed. To minimize confounding effects arising from differences in fermentation characteristics, samples with similar fermentation quality were selected prior to phenethyl acetate application. This allowed for a more accurate assessment of the compound's influence on intake preference. Therefore, this study investigated the effects of spraying varying concentrations of phenethyl acetate on the feeding behavior of high-quality fermented alfalfa with comparable fermentation profiles. No significant differences were found in fermented alfalfa average intake, average DM intake, feed preference, feeding rate, or intake rate between the LP and LPPL groups; however, significant (*P* < 0.001) differences were identified in these variables between the LP and LPPH groups (Fig. 8A-E). Compared with the LP group (Supplementary Table 2–3), the LPPL group exhibited a feed preference rate of 47.70 % (< 50 %), whereas the LPPH group showed a significantly (*P* < 0.001) higher feed preference rate of 56.46 % (> 50 %). These results suggest that sheep preferred the fermented alfalfa from the LPPH group, but not that from the LPPL group. Overall, the findings indicate that a high concentration of phenethyl acetate (50 mg/kg) was associated with increased feed intake and improved feeding behavior in sheep. In contrast, a low concentration of phenethyl acetate (5 mg/kg) does not exert such an effect. At lower concentrations, phenethyl acetate may reduce the preference, indicating that the effect on palatability is non-linear and dose-dependent. One possible explanation is that phenethyl acetate may have an optimal concentration range for maximizing feed intake, consistent with the “inverted U-shaped” response often observed in olfactory stimuli (Provenza, 1995). Below this threshold, the aroma may be insufficient to stimulate interest or may even be perceived as unnatural or unpalatable when not balanced with other volatile compounds. Alternatively, the addition of phenethyl acetate—even at low levels—might disrupt the natural volatile profile of the silage, leading to a perceived imbalance in odor complexity that sheep instinctively avoid. This phenomenon highlights the importance of dose optimization in flavor-based feed additives. Future studies should systematically evaluate concentration gradients of phenethyl acetate to identify the threshold for positive preference and assess potential interactions with other fermentation-derived volatiles.

These short-term feeding preference results highlight that phenethyl acetate plays an important role in the feeding behavior of sheep. And this short-term preference test is well supported by previous studies. Scherer et al. (2019) demonstrated that 30-min and 3-h feeding preferences are highly consistent, as the silage identification and decision-making process was already underway in the first few minutes based on the odor emitted by the silage. Similarly, Scherer et al. (2021), reported that concentrations of high-quality flavor substances, such as 2-carboxy-4-dodecanolide with a fruity aroma and fructose with a sweet taste, were higher in fermented forages with high feed intake. Ahmad et al. (2024) found that the use of flavoring additives caused some changes in the feeding pattern of newly received steers. Martí et al. (2023) reported that, in unweaned dairy beef calves, the intake of concentrate supplemented with sensory additives increased between days 28 and 42. Therefore, phenethyl acetate (50 mg/kg) can be used as a target metabolite to enhance the flavor profile of fermented alfalfa, potentially increasing the feed intake of ruminants and may become an attractant for ruminants in animal husbandry production. *L. plantarum* B90 has the potential to be a functional strain for the production of ruminant attractants.

Feed flavor and post-ingestive feedback are key regulators of ruminant feeding behavior (Provenza et al., 1996). While phenethyl acetate likely acts as an olfactory attractant, its influence on taste perception or feed nutritional dynamics remains unclear. Future studies should investigate the neural, sensory, and metabolic pathways involved to fully understand how specific aroma compounds influence ruminant feeding behavior. In addition, *L. plantarum* B90 shows promise as a functional additive for improving both silage fermentation and feed palatability. From a practical standpoint, its application in large-scale production depends on cost-effective fermentation and formulation processes, which are feasible given established probiotic manufacturing practices. Alternatively, incorporating phenethyl acetate as an additive during forage fermentation may offer another strategy to enhance feeding behavior and warrants further investigation. However, for both approaches, the stability of the active components (the strain and phenethyl acetate) during storage and processing, as well as their long-term effects on ruminants' health and feeding behavior, requires rigorous evaluation before widespread use can be recommended.

## 4 Conclusion

In summary, the addition of L. *plantarum* B90 significantly improved the fermentation quality of fermented alfalfa and increased the proportion of esters and aldehydes. As a core microorganism in fermented alfalfa, *L. plantarum* utilizes phenylacetaldehyde to synthesize phenylethyl alcohol, which serves as a precursor to the key flavor compound phenethyl acetate. Additionally, fermented alfalfa sprayed with phenethyl acetate (50 mg/kg) was associated with increased feed intake in sheep. These findings highlight the potential of L. *plantarum* B90 to improve the flavor quality of fermented alfalfa, while also demonstrating the effectiveness of phenethyl acetate as an attractive metabolite for ruminants. This paves the way for developing high-quality additives that improve fermented forage flavor and promote feed intake in ruminants.

## CRediT authorship contribution statement

**Zhihui Fu:** Writing – original draft, Formal analysis, Data curation. **Tianwei Wang:** Writing – review & editing, Funding acquisition. **Jiaqi Zhang:** Data curation. **Wenzhao Wang:** Investigation. **Xiumin Zhang:** Investigation. **Muhammad Tahir:** Supervision. **Jin Zhong:** Supervision, Funding acquisition.

## Declaration of competing interest

The authors declare that they have no known competing financial interests or personal relationships that could have appeared to influence the work reported in this paper.

## Data Availability

Data will be made available on request.

## Appendix A Supplementary data

Supplementary material Image 1

## References

[bb0005] Ahmad M., Seddon Y.M., Blanch M., Penner G.B., Moya D. (2024). Effects of flavoring additives on feed intake, growth performance, temperament, and markers of immune function for newly received feedlot cattle. Journal of Animal Science.

[bb0010] Fan X., Du L., Xu J., Shi Z., Zhang T., Jiang X., Zeng X., Wu Z., Pan D. (2022). Effect of single probiotics *Lacticaseibacillus casei* CGMCC1.5956 and *Levilactobacillus brevis* CGMCC1.5954 and their combination on the quality of yogurt as fermented milk. LWT- Food Science and Technology.

[bb0015] Fu Z.H., Sun L., Hou M.L., Hao J.F., Lu Q., Liu T.Y., Ren X.Z., Jia Y.S., Wang Z.J., Ge G.T. (2022). Effects of different harvest frequencies on microbial community and metabolomic properties of annual ryegrass silage. Frontiers in Microbiology.

[bb0020] Guo T., Liu X., Li F., Lv J.R., Liu F., Hang M.Y., Zhang Z.C. (2022). Effects of different attractants on the feeding preferences, growth, and blood parameters in the fattening of Hu sheep. Pratacultural Science.

[bb0025] Hafner S.D., Howard C., Muck R.E., Franco R.B., Montes F., Green P.G., Mitloehner F., Trabue S.L., Rotz C.A. (2013). Emission of volatile organic compounds from silage: Compounds, sources, and implications. Atmospheric Environment.

[bb0030] Huang F.Q., Wang T.W., Zhang J.Q., Tahir M., Sun J.H., Liu Y.Y., Yun F.F., Xia T.Q., Teng K.L., Wang J.W., Zhong J. (2023). Exploring the bacterial community succession and metabolic profiles of *Lonicera japonica* Thunb residues during anaerobic fermentation. Bioresource Technology.

[bb0035] Keranmu A., Pan L.B., Fu J., Han P., Yu H., Zhang Z.W., Xu H., Yang X.Y., Hu J.C., Zhang H.J., Bu M.M., Jiang J.D., Xing N.Z., Wang Y. (2022). Biotransformation of liquiritigenin into characteristic metabolites by the gut microbiota. Molecules.

[bb0040] Lawson C.E., Wu S., Bhattacharjee A.S., Hamilton J.J., McMahon K.D., Goel R., Noguera D.R. (2017). Metabolic network analysis reveals microbial community interactions in anammox granules. Nature Communications.

[bb0045] Liang T., Jiang T., Liang Z., Zhang N., Dong B., Wu Q., Gu B. (2023). Carbohydrate-active enzyme profiles of *Lactiplantibacillus plantarum* strain 84-3 contribute to flavor formation in fermented dairy and vegetable products. Food Chemistry: X.

[bb0050] Liao C., Na B., Tang X., Zhao M., Zhang C., Chen S., Li P. (2023). Contribution of the bacterial community of poorly fermented oat silage to biogas emissions on the Qinghai Tibetan plateau. Science of the Total Environment.

[bb0055] Liu C., Du M.X., Xie L.S., Wang W.Z., Chen B.S., Yun C.Y., Sun X.W., Luo X., Jiang Y., Wang K., Jiang M.Z., Qiao S.S., Sun M., Cui B.J., Huang H.J., Qu S.P., Li C.K., Wu D., Wang L.S., Liu S.J. (2024). Gut commensal Christensenella minuta modulates host metabolism via acylated secondary bile acids. Nature Microbiology.

[bb0060] Liu M.J., Siezen R. (2006). Comparative genomics of flavour-forming pathways in lactic acid bacteria. Australian Journal of Dairy Technology.

[bb0065] Liu S.P., Chen Q.L., Zou H.J., Yu Y.J., Zhou Z.L., Mao J., Zhang S. (2019). A metagenomic analysis of the relationship between microorganisms and flavor development in Shaoxing mechanized huangjiu fermentation mashes. International Journal of Food Microbiology.

[bb0070] Liu Y., Du S., Sun L., Li Y., Liu M., Sun P., Bai B., Ge G., Jia Y., Wang Z. (2024). Volatile metabolomics and metagenomics reveal the effects of lactic acid bacteria on alfalfa silage quality, microbial communities, and volatile organic compounds. Communications Biology.

[bb0075] Man L., Ren W., Qin H., Sun M., Yuan S., Zhu M., Liu G., Wang C., Li M. (2023). Characterization of the relationship between lipids and volatile compounds in donkey, bovine, and sheep meat by UHPLC–ESI–MS and SPME–GC–MS. LWT- Food Science and Technology.

[bb0080] Manzocchi E., Martin B., Bord C., Verdier-Metz I., Bouchon M., De Marchi M., Constant I., Giller K., Kreuzer M., Berard J., Musci M., Coppa M. (2021). Feeding cows with hay, silage, or fresh herbage on pasture or indoors affects sensory properties and chemical composition of milk and cheese. Journal of Dairy Science.

[bb0085] Martí S., Blanch M., Devant M. (2023). Use of feed additives added to the milk replacer and concentrate or only to the concentrate in unweaned dairy beef calves after transportation and its effects on performance. Journal of Animal Science.

[bb0090] Nguyen N.T.H., Wang W.-Y., Huang W.-L., Huang C.-L., Chiang T.-Y. (2022). Metagenomics analyses of microbial dynamics associated with putative flavor development in mash fermentation of sake. LWT- Food Science and Technology.

[bb0095] Patrica C. (1997).

[bb0100] Provenza F.D. (1995). Postingestive feedback as an elementary determinant of food preference and intake in ruminants. Journal of Range Management.

[bb0105] Provenza F.D., Scott C.B., Phy T.S., Lynch J.J. (1996). Preference of sheep for foods varying in flavors and nutrients. Journal of Animal Science.

[bb0110] Rao C., Coyte K.Z., Bainter W., Geha R.S., Martin C.R., Rakoff-Nahoum S. (2021). Multi-kingdom ecological drivers of microbiota assembly in preterm infants. Nature.

[bb0115] Ricci M., Gasperi F., Betta E., Menghi L., Endrizzi I., Cliceri D., Franceschi P., Aprea E. (2023). Multivariate data analysis strategy to monitor Trentingrana cheese real-scale production through volatile organic compounds profiling. LWT- Food Science and Technology.

[bb0120] Scherer R., Gerlach K., Ghaffari M.H., Südekum K.H. (2021). Linking forage choice behavior of goats with the metabolome of contrasting silages. Journal of Dairy Science.

[bb0125] Scherer R., Gerlach K., Südekum K.H. (2019). Decision-making of goats when exposed to choice feeding: Triggered by taste or smell?. Applied Animal Behaviour Science.

[bb0130] Sekar B.S., Li X., Li Z. (2022). Bioproduction of natural phenethyl acetate, phenylacetic acid, ethyl phenylacetate, and phenethyl phenylacetate from renewable feedstock. ChemSusChem.

[bb0135] Tan X., Yang Y.L., Liu Y.W., Li X., Zhu W.B. (2021). Quantitative ecology associations between heterotrophic nitrification-aerobic denitrification, nitrogen-metabolism genes, and key bacteria in a tidal flow constructed wetland. Bioresource Technology.

[bb0140] Tatli Seven P., Yildirim E.N., Seven I., Kaya C.A., Iflazoglu Mutlu S. (2024). An evaluation of the effectiveness of sumac and molasses as additives for alfalfa silage: Influence on nutrient composition, in vitro degradability and fermentation quality. Journal of Animal Physiology and Animal Nutrition.

[bb0145] Van S.P.V., Robertson J.B., Lewis B.A. (1991). Methods for dietary fiber, neutral detergent fiber, and nonstarch polysaccharides in relation to animal nutrition. Journal of Dairy Science.

[bb0150] Wang C., He L., Xing Y., Zhou W., Yang F., Chen X., Zhang Q. (2019). Fermentation quality and microbial community of alfalfa and stylo silage mixed with Moringa oleifera leaves. Bioresource Technology.

[bb0155] Wang D., Chen G., Tang Y., Li J., Huang R., Ye M., Zhang W. (2022). Correlation between autochthonous microbial communities and flavor profiles during the fermentation of mustard green paocai (*Brassica juncea* Coss.), a typical industrial-scaled salted fermented vegetable. *Lwt-food*. Science and Technology.

[bb0160] Wang Y., Chen X.Y., Wang C., He L.W., Zhou W., Yang F.Y., Zhang Q. (2019). The bacterial community and fermentation quality of mulberry (*Morus alba*) leaf silage with or without and sucrose. Bioresource Technology.

[bb0165] Xia T., Tahir M., Wang T., Wang Y., Zhang X., Liu S., Teng K., Fu Z., Yun F., Wang S., Jin S., Hu J., Zhong J. (2024). *Lactobacillus cocktail* and cellulase synergistically improve the fiber transformation rate in *Sesbania cannabina* and sweet sorghum mixed silage. *Chemical and Biological Technologies in* Agriculture.

[bb0170] Xiao Y., Liu Y., Chen C., Xie T., Li P. (2020). Effect of *Lactobacillus plantarum* and *staphylococcus xylosus* on flavour development and bacterial communities in Chinese dry fermented sausages. Food Research International.

[bb0175] Xiong S., Xu X., Zhang L., Du T., Huang T., Huang J., Ren H., Xiong T., Xie M. (2024). Integrated metatranscriptomics and metabolomics reveal microbial succession and flavor formation mechanisms during the spontaneous fermentation of Laotan Suancai. Food Research International.

[bb0180] Yang Y., Li J., Xing J., Xing W., Tang C., Rao Z., Zhang J. (2022). Untargeted profiling and differentiation of volatiles in varieties of meat using GC orbitrap MS. Foods.

[bb0185] Yang Y., Wang S.T., Lu Z.M., Zhang X.J., Chai L.J., Shen C.H., Shi J.S., Xu Z.H. (2021). Metagenomics unveils microbial roles involved in metabolic network of flavor development in medium-temperature daqu starter. Food Research International.

[bb0190] Yang Z.J., Wang D., Li Y.Y., Zhou X.F., Liu T.T., Shi C., Wu X.H. (2022). Untargeted metabolomics analysis of the anti-diabetic effect of red ginseng extract in type 2 diabetes mellitus rats based on UHPLC-MS/MS. Biomedicine & Pharmacotherapy.

[bb0195] Yao H.B., Su H., Ma J.Y., Zheng J., He W., Wu C.L., Hou Z.Y., Zhao R.L., Zhou Q.Q. (2023). Widely targeted volatileomics analysis reveals the typical aroma formation of Xinyang black tea during fermentation. Food Research International.

[bb0200] Yu H., Li Z., Zheng D., Chen C., Ge C., Tian H. (2024). Exploring microbial dynamics and metabolic pathways shaping flavor profiles in Huangjiu through metagenomic analysis. Food Research International.

[bb0205] Zhang B., Xu D., Duan C., Yan G. (2020). Synergistic effect enhances 2-phenylethyl acetate production in the mixed fermentation of Hanseniaspora vineae and Saccharomyces cerevisiae. Process Biochemistry.

[bb0210] Zhang Q., Guo X., Zheng M.Y., Chen D.K., Chen X.Y. (2021). Altering microbial communities: A possible way of lactic acid bacteria inoculants changing smell of silage. Animal Feed Science and Technology.

[bb0215] Zhang X., Usman S., Bature I., Xu D.M., Guo X.S. (2023). Occurrence and fate of antibiotic-resistance genes and their potential hosts in high-moisture alfalfa silage treated with or without formic acid bactericide. Journal of Environmental Management.

[bb0220] Zhao Y., Wang Y.Q., Li C.S., Li L.H., Yang X.Q., Wu Y.Y., Chen S.J., Zhao Y.Q. (2021). Novel insight into physicochemical and flavor formation in naturally fermented tilapia sausage based on microbial metabolic network. Food Research International.

