# Supplementary material for: Multi-omics profiling reveals microbial regulation of a key aromatic ester phenethyl acetate formation in fermented alfalfa and its impact on sheep feed preference
Source: Food Chem X. 2025 Nov 4;32:103249. doi: 10.1016/j.fochx.2025.103249 (PMC12639630; doi:10.1016/j.fochx.2025.103249)
Supplement: Supplementary file 1 — Supplementary material [file mmc1.docx]

**Supplementary Table 1** Effects of low concentration phenethyl acetate treated alfalfa silage on feed preference and feed intake of sheep

| Items | LP | SEM |
| --- | --- | --- |
| **Nutritional quality** |  |  |
| DM, % | 39.76 | 0.18 |
| CP, %DM | 19.85 | 0.05 |
| ADF, %DM | 32.50 | 0.23 |
| NDF, %DM | 37.40 | 0.19 |
| WSC, %DM | 2.98 | 0.20 |
| **Fermentation quality** |  |  |
| pH | 4.46 | 0.02 |
| LA, %DM | 10.79 | 0.08 |

DM: dry matter; CP: crude protein; ADF: acid detergent fiber; NDF: neutral detergent fiber; WSC: water soluble carbohydrate; pH: pH value; LA: lactic acid; LP: *Lactiplantibacillus plantarum* B90 additives. SEM: standard error of mean.

**Supplementary Table 2** Effects of low concentration phenethyl acetate sprayed alfalfa silage on feed behavior of sheep

| Attribute | Treatment | | SEM | *P* value |
| --- | --- | --- | --- | --- |
|  | LP | LPPL |  |  |
| Average intake, g/d | 1079.17 | 967.82 | 82.90 | 0.0611 |
| Average DM intake, g/d | 429.08 | 384.81 | 32.96 | 0.0611 |
| Feeding rate, g/min | 17.99 | 16.13 | 1.38 | 0.0612 |
| Intake rate, % | 52.01 | 46.70 | 4.03 | 0.0642 |
| Feed preference, % | 52.23 | 47.70 | 1.51 | 0.0668 |

LP: Alfalfa silage fermented by *Lactiplantibacillus plantarum* B90 served as the control group. LPPL: A low concentration (5 mg/kg) of phenethyl acetate was sprayed on the silage in the LP group, which served as the test group LPPL. SEM: standard error of mean.

**Supplementary Table 3** Effects of high concentration phenethyl acetate sprayed alfalfa silage on feed behavior of sheep

| Attribute | Treatment | | SEM | *P* value |
| --- | --- | --- | --- | --- |
|  | LP | LPPH |  |  |
| Feed intake, g/d | 898.33 | 1186.25***** | 90.83 | 0.0007 |
| DM feed intake, g/d | 357.18 | 471.66***** | 36.11 | 0.0007 |
| Feeding rate, g/min | 14.97 | 19.77***** | 1.51 | 0.0007 |
| Intake rate, % | 43.23 | 57.07***** | 4.36 | 0.0006 |
| Feed preference, % | 43.53 | 56.46***** | 3.52 | 0.0001 |

LP: Alfalfa silage fermented by *Lactiplantibacillus plantarum* B90 served as the control group. LPPH: A high concentration (50 mg/kg) of phenethyl acetate was sprayed on the silage in the LP group, which served as the test group LPPH. SEM: standard error of mean. **^*^**Mean values differ compared with the control at *P* < 0.001.
